# Concurrent Validity of the Adult Eating Behavior Questionnaire in a Canadian Sample

**DOI:** 10.3389/fpsyg.2021.779041

**Published:** 2021-12-02

**Authors:** Tamara R. Cohen, Lisa Kakinami, Hugues Plourde, Claudia Hunot-Alexander, Rebecca J. Beeken

**Affiliations:** ^1^Faculty of Land and Food Systems, University of British Columbia, Vancouver, BC, Canada; ^2^PERFORM Centre, Concordia University, Montreal, QC, Canada; ^3^Department of Mathematics and Statistics, Concordia University, Montreal, QC, Canada; ^4^School of Human Nutrition, McGill University, Sainte-Anne-de-Bellevue, QC, Canada; ^5^Instituto de Nutrición Humana, Departamento de Reproducción Humana, Crecimiento y Desarrollo Infantil, Centro Universitario de Ciencias de la Salud, Universidad de Guadalajara, Guadalajara, Mexico; ^6^Leeds Institute of Health Sciences, University of Leeds, Leeds, United Kingdom; ^7^Department of Behavioural Science and Health, University College London, London, United Kingdom

**Keywords:** eating behaviours, appetitive traits, validation, questionnaire, psychometrics, adult, overweight, obesity

## Abstract

The current study aimed to test the factor structure of the Adult Eating Behavior Questionnaire (AEBQ), its construct validity against the Three-Factor Eating Questionnaire (TFEQ-R18) and its associations with body mass index (BMI) in Canadian adults (*n* = 534, 76% female). Confirmatory factor analysis (CFA) revealed that a seven-factor AEBQ model, with the Hunger subscale removed, had better fit statistics than the original eight-factor structure. Cronbach’s alpha was used to assess the internal reliability of each subscale and resulted with α > 0.70 for all subscales except for Hunger (α = 0.68). Pearson’s correlations were used to inform the convergent and discriminant validation of AEBQ against the TFEQ-R18 and to examine the relationship between AEBQ and BMI. All AEBQ Food Approach subscales positively correlated with that of the TFEQ-R18 Emotional Eating and Uncontrolled Eating subscales. Similarly, BMI correlated positively with Food Approach subscales (except Hunger) and negatively with Food Avoidance subscales (except Food Fussiness). These results support the use of a seven-factor AEBQ for adults self-reporting eating behaviors, construct validity of the AEBQ against TFEB-R18, and provide further evidence for the association of these traits with BMI.

## Introduction

Overweight and obesity remain a global public health concern (World Health Organization [WHO], 2000). In Canada, it is estimated that the adult Canadian population has body mass indices (BMI) that classify 36% of them as living with an overweight condition and 27% of them living with obesity (Government of Canada, 2018). Overweight and obesity conditions are risk factors for a variety of non-communicable diseases such as diabetes mellitus, cardiovascular diseases, hypertension, and certain types of cancer (Nyberg et al., 2018). The prevalence and impact of weight and obesity indicate the importance of examining effective prevention and treatment strategies as they relate to health behaviors such as eating behaviors. Results from a previous review have shown that certain eating behaviors are positively associated with increased body mass index (BMI) and obesity (French et al., 2012). For instance, subscales from the Adult Eating Behaviour Questionnaire (AEBQ) have been shown to be associated with BMI in the expected direction, such as higher Food Approach subscales and lower Food Avoidance subscales were positively associated with individuals living with higher BMIs (Hunot et al., 2016).

Eating behaviors are often described as appetitive traits, which are genetic predispositions towards food that interact with environmental factors to influence eating behaviors (Carnell et al., 2013). While certain AEBQ subscales are associated with body weight and other measures of adiposity, studies in children have demonstrated that the traits captured by the tool are also associated with a range of other important factors such as food preferences (Fildes et al., 2015), dietary patterns (Carnell et al., 2016), sleep (Miller et al., 2019), and cardiometabolic health (Warkentin et al., 2020). Appetitive traits are conventionally assessed using questionnaires, such as Healthy Eating Index, Three-Factor Eating Questionnaire, and Self-Regulation of Eating Behavior Questionnaire (Karlsson et al., 2000; Guenther et al., 2008; Kliemann et al., 2016). The AEBQ is a 35-item questionnaire that assesses appetitive traits (Hunot et al., 2016). Unlike other questionnaires, the AEBQ includes eight subscales within two categories of eating behaviors (*Food Approach:* Food Responsiveness, Hunger, Emotional Overeating, and Enjoyment of Food; *Food Avoidance:* Satiety Responsiveness, Food Fussiness, Emotional Undereating, and Slowness in Eating).

The AEBQ was intentionally modeled off the Children’s Eating Behaviour Questionnaire (CEBQ) (Wardle et al., 2001) and was adapted to an adults’ eating behavior by including “Hunger” and “Food responsiveness” subscales (Wardle et al., 2001; Hunot et al., 2016). However, Hunot et al. (2016) from the original publication acknowledged that the interpretation of the AEBQ Hunger subscale may differ between individuals and therefore additional validation studies would be needed to examine the validity of the AEBQ (Hunot-Alexander et al., 2019). Further, authors found that the Hunger and Food Responsiveness subscales overlapped, and therefore questioned whether the Hunger subscale should be combined with Food Responsiveness subscales, or whether they should remain as separate subscales (Hunot et al., 2016). Despite some suggesting a better fitting model when the questionnaire included the original 8-factors (Mallan et al., 2017; Zickgraf and Rigby, 2019; Jacob et al., 2021), others have found improved reliability estimates when the Hunger scale is dropped from the model and not combined with Food Responsiveness (Mallan et al., 2017; Hunot-Alexander et al., 2019). Additional studies that further contribute to these inconsistencies are needed.

To date, AEBQ validation studies have been limited to five studies with adult samples (Australia, United States, Bulgaria, Mexico, and Canada) (Mallan et al., 2017; Hristova, 2019; Zickgraf and Rigby, 2019; Hunot-Alexander et al., 2021a; Jacob et al., 2021), one with younger adults, and three with adolescent samples (Chinese, United Kingdom, Polish) (Hunot-Alexander et al., 2019; Guzek et al., 2020; He et al., 2021). To our knowledge, the AEBQ has not been validated in an English Canadian sample. Similarly, to our knowledge, there are no reports comparing the scores of the AEBQ to that of existing measures of eating behaviors (such as the TFEQ-R18) to test for associations between similar subscales in an English Canadian sample.

The aims of this study were first and primarily to confirm the fit statistics of different AEBQ models, second to test the convergent and discriminant validity between the scores from the AEBQ and the TFEQ-R18, and third to examine the associations between AEBQ subscale scores and self-reported BMI in a sample of healthy Canadian adults. We hypothesize the following: (1) the 7-factor AEBQ (excluding Hunger subscale) would show a significantly better fit compared to the original 8-factor structure; (2) the AEBQ would demonstrate convergent and discriminant validity against the TFEQ-R18, whereby all Food Approach scores would positively correlate with the Uncontrolled Eating and Emotional Eating TFEQ-R18 subscales; and (3) the AEBQ Food Approach subscales will positively correlate and the Food Avoidance subscales will negatively correlate with BMI.

## Materials and Methods

### Procedure

Participants were recruited (May to September 2018) from social media platforms through the PERFORM Centre (Concordia University) (e.g., e-mail Listserv (>3000 members), Facebook, and Instagram). The PERFORM Centre Listserv includes students, staff, and faculty members of Concordia University as well as members of the general public who have signed up to receive their newsletter. Eligibility included being 18 years and older, are psychologically healthy, with no current or previous history of a diagnosed eating disorder(s). Due to the nature of recruitment and sampling, estimates of response rates were not possible.

The survey was in English and took approximately 10–15 min to complete. At the end of the survey, participants had the option to enter their e-mail address and first name into a raffle to win a Samsung Galaxy Tablet (valued at $200 CAD). To maintain the anonymous nature of the survey, their e-mail address was not linked to their results. The winner was chosen at random. This study was approved by the Concordia Research Ethics Board (30009490). All participants provided informed consent.

### Sample

Seven hundred and thirty-three individuals consented to participate and started the one-time anonymous online survey. Data collected included the AEBQ, TFEQ-R-18, and self-reported demographic information (date of birth, height, weight, sex, education, and ethnicity). BMI was calculated based on self-reported weight (kg) divided by self-reported height (m)-squared. Overall, 568 participants completed the survey but 30 did not report their age or were less than 18 years of age, and four did not self-report either height or weight and, thus, were excluded from the analysis, resulting in a final sample of 534 participants. These participants who were excluded (*n* = 34) did not differ from the full analytic sample in sex, education level, weight status, or AEBQ subscale means (data not shown). Participant mean age was 39.5 ± 16.4 years (Table 1). The majority had completed a high school education (96%) and were identified as Caucasian (70%) females (76%). Mean BMI was 24.9 ± 5.1 kg/m^2^, with 56% classified as having a normal BMI (i.e., <24.9 kg/m^2^) as per World Health Organization BMI classifications (Weir and Jan, 2021).

**TABLE 1:** Characteristics of participants who completed all components of the online questionnaire (*n* = 534).

	*n*	%
**Age**		
18–35 y	279	52.2
36–55 y	145	27.1
>55 y	110	20.6
**Sex**		
Female	404	75.7
Male	130	24.3
**Ethnicity**		
White	375	70.2
Black	14	2.6
Asian	45	8.4
Hispanic	24	4.5
Other	76	14.2
**Education**		
At least some college	511	96.4
High school or less	19	3.6
**BMI classification**		
Underweight	21	3.9
Normal	301	56.4
Overweight	133	24.9
Obese	79	14.8

### Measures

#### Adult Eating Behavior Questionnaire

The AEBQ is a 35-item questionnaire that assesses “Food Approach” and “Food Avoidance” subscales. Food Approach subscales include Food Responsiveness (four items, e.g., “*When I see or smell food that I like, it makes me want to eat it”)*, Hunger (five items, e.g., “*If my meals are delayed I get light-headed*”), Emotional Overeating (five items, “*I eat more when I’m upset*”), and Enjoyment of Food (three items, e.g., “*I love food*”) (Hunot et al., 2016). Food Avoidance subscales include Satiety Responsiveness (four items, e.g., “*I often get full before my meal is finished*”), Food Fussiness (five items, e.g., “*I often decide that I don’t like a new food before tasting it*”), Emotional Undereating (five items, e.g., “*I eat less when I’m upset*”), and Slowness in Eating (four items, e.g., “*I am often last at finishing a meal*”) (Hunot et al., 2016). Participants rated their responses using a 5-point Likert scale from ‘1 = Strongly Disagree” to “5 = Strongly Agree.” The mean scores of each subscale were calculated as per the original publication.

#### Three-Factor Eating Questionnaire

The TFEQ-R18 is a 18-item questionnaire that assesses three different aspects of eating behavior: Cognitive restraint, or restrained eating (6 items, e.g., *“I deliberately take small helpings to control my weight”*), Uncontrolled Eating (nine items, e.g., *“Sometimes when I start eating, I just can’t seem to stop.”*) and Emotional Eating (three items, e.g., *“I start to eat when I feel anxious.”*). Participants responded to questions using a one to four score system [Definitely false (1), mostly false (2), mostly true (3), and definitely true (4)] (Karlsson et al., 2000). Scoring of questions were completed as per questionnaire instructions (Karlsson et al., 2000). Permission to use the questionnaire in this study was sought and granted from authors. Karlsson et al. (2000) reported Cronbach’s alphas for each of the three aspects were above 0.70 but below 0.90. Psychometric properties of the TFEQ-R18 has been validated in many populations and adapted (e.g., Spanish (Jáuregui-Lobera et al., 2014), Persian (Mostafavi et al., 2017), Greek (Kavazidou et al., 2012), Finnish (Anglé et al., 2009), and French (Lauzon et al., 2004), among others), all supporting the reliability and validity of the tool for use in research studies and clinical practice.

### Statistical Analysis

All analyses were conducted with SAS 9.4 (SAS Institute, Cary, NC, United States). Confirmatory Factor Analysis (CFA) with maximum likelihood estimation was used to test alternative models of the AEBQ based on Hunot et al and Mallan et al. (Hunot et al., 2016; Mallan et al., 2017). Model 1 included all 35 questions (8-factors) from the original AEBQ (Hunot et al., 2016). Model 2 included 35 questions but 7-factors, with Food Responsiveness and Hunger merged to one subscale. Model 3 included 30 questions (7-factors), with the Hunger subscale removed from the analysis. Models were evaluated for goodness of fit using the following indices: comparative Fit Index (CFI, where values >0.90 suggest a good model fit), Goodness of Fit Index (GFI, where values >0.90 suggest a good model fit), Root Mean Square Error of Approximation (RMSEA, with values are ideally ≤0.06), and Akaike’s Information Criteria (AIC, used to compare models whereby the smaller AIC is preferred (Cavanaugh and Neath, 2019).

Descriptive statistics were used to describe participants. General lineal models (GLM) were used to assess for differences among age groups (18–35 y, 36–55 y, and 55 + y) adjusting for BMI category (underweight/normal versus overweight/obese) and sex and for differences among BMI categories (underweight/normal versus overweight/obese) adjusting for age and sex. Given there were only *n* = 21 underweight individuals, and the fact that weight and height were self-reported, we decided to merge this BMI category with normal weight. The mean for each subscale was calculated. Cronbach’s alpha was used to assess the internal reliability of each subscale. Pearson’s correlations were used to inform the convergent and discriminant validation with the TFEQ-R18 and to examine the relationships between the AEBQ and participant’s BMI (adjusted for sex and age). Data was also analyzed as “unadjusted” but did not differ from adjusted therefore we report on adjusted data.

## Results

### Factor Structure of Adult Eating Behavior Questionnaire Subscales

Cronbach’s alphas and mean subscale scores from the AEBQ are presented in Table 2. The Confirmatory Factor Analysis (CFA) is presented in Table 3. Cronbach’s alpha values were all >0.70 except for Hunger scores (Cronbach’s α = 0.68). Mean scores for Food Approach subscales were generally higher (total mean score: 3.37 ± 0.54) than Food Avoidance subscales (total mean score: 2.44 ± 0.48). CFA was used to compare the full 8-subscale AEBQ (Model 1) with the two alternative 7-subscale models (Model 2 and Model 3). Our results suggest that all three models showed a relatively decent model fit (defined as having chi-square/degree of freedom <3.0; CFI values >0.90 and RMSEA values 0.05–0.10) (Hu and Bentler, 1999). AIC values suggest that the fit statistics were improved when the Hunger subscale was removed from the analysis: a finding that was suggested by Hunot et al. and later echoed by Mallan et al. (Hunot et al., 2016; Mallan et al., 2017; Table 4).

**TABLE 2:** Internal consistency estimates (Cronbach’s α) and mean scores (mean ± standard deviation) from the original 8-subscales and the new variable (Food Responsiveness + Hunger) by body mass index classification and age group.

	Cronbach’s α	Total Sample (*n* = 534)	Body Mass Index				Age				
Variable			Underweight/Normal (*n* = 322)	Overweight/Obese (*n* = 212)	Unadjusted *p*-value	Adjusted (by age and sex) *p*-value	18–35 y (*n* = 279)	36–55 y (*n* = 145)	>56 + y (*n* = 110)	Unadjusted *p*-value	Adjusted (by BMI and sex) *p*-value
Enjoyment of food (*n* = 3 items)	0.83	4.26 ± 0.76	4.25 ± 0.77	4.26 ± 0.75	0.698	0.316	4.36 ± 0.74	4.26 ± 0.70	3.98 ± 0.81^a^	<0.001	<0.001
Emotional overeating (*n* = 5 items)	0.90	2.90 ± 1.02	2.82 ± 0.78	3.14 ± 0.78	<0.001	<0.001	2.95 ± 0.76	3.04 ± 8.44	2.82 ± 0.83^b^	0.092	0.058
Food responsiveness (*n* = 4 items)	0.73	3.22 ± 0.78	3.12 ± 0.79	3.26 ± 0.74	0.600	<0.001	3.56 ± 0.75	3.08 ± 0.71^c^	2.85 ± 0.67^d^	<0.001	<0.001
Hunger (*n* = 5 items)	0.68	3.13 ± 0.72	3.17 ± 0.68	3.13 ± 0.65	0.440	0.538	3.30 ± 0.66	3.10 ± 0.61^e^	2.83 ± 0.66^f^	<0.001	<0.001
Emotional undereating (*n* = 5 items)	0.88	2.81 ± 0.89	2.85 ± 0.67	2.64 ± 0.63	0.338	<0.001	2.88 ± 0.67	2.64 ± 0.65^g^	2.63 ± 0.63	<0.001	0.046
Food fussiness (*n* = 5 items)	0.88	1.99 ± 0.79	1.96 ± 0.78	2.03 ± 0.80	0.664	0.664	1.99 ± 0.80	1.97 ± 0.82	2.01 ± 0.73	0.927	0.941
Slowness in eating (*n* = 4 items)	0.88	2.58 ± 0.98	2.72 ± 0.97	2.35 ± 0.95	0.680	0.0001	2.59 ± 1.03	2.55 ± 0.86	2.60 ± 1.01	0.929	0.550
Satiety responsiveness (*n* = 4 items)	0.72	2.46 ± 0.74	2.51 ± 0.74	2.83 ± 0.72	0.618	0.389	2.47 ± 0.79	2.40 ± 0.66	2.48 ± 0.72	0.593	0.579
Food responsiveness and hunger combined	0.77	3.17 ± 0.63	3.15 ± 0.65	3.18 ± 0.62	0.345	0.005	3.33 ± 0.62	3.09 ± 0.57^h^	2.84 ± 0.59^i^	0.0002	<0.001

*^a^versus 18–35 y, adjusted p < 0.001; ^b^versus 18–35 y, adjusted p = 0.025; ^c^versus 18–35 y, adjusted p < 0.001; ^d^versus 18–35 y, adjusted p < 0.001; ^e^versus 18–35 y, adjusted p = 0.013; ^f^versus 18–35 y, adjusted p < 0.001; ^g^versus 36–55 y, adjusted p = 0.029; ^h^versus 18–35 y, adjusted p < 0.001; ^i^versus 18–35 y, adjusted p < 0.001.*

**TABLE 3:** Factor loading of the 35 item AEBQ (*n* = 534).

Factors	Item number	Items	Factor loadings		
			Estimate		Standard error
Hunger					
	6	I often notice my stomach rumbling	0.35	+	0.04
	9	If I miss a meal, I get irritable	0.51	+	0.04
	28	I often feel so hungry that I have to eat something right away	0.66	+	0.03
	32	I often feel hungry	0.69	+	0.03
	34	If my meals are delayed, I get light-headed	0.49	+	0.04
Food responsiveness					
	13	I often feel hungry when I am with someone who is eating	0.57	+	0.03
	17	Given the choice, I would eat most of the time	0.75	+	0.03
	22	I am always thinking about food	0.73	+	0.03
	33	When I see or smell food that I like, it makes me want to eat	0.49	+	0.04
Emotional overeating					
	5	I eat more when I’m annoyed	0.76	+	0.02
	8	I eat more when I’m worried	0.86	+	0.01
	10	I eat more when I’m upset	0.87	+	0.01
	16	I eat more when I’m anxious	0.80	+	0.02
	21	I eat more when I’m angry	0.75	+	0.02
Enjoyment of food					
	1	I love food	0.69	+	0.03
	3	I enjoy eating	0.81	+	0.02
	4	I look forward to mealtimes	0.87	+	0.02
Satiety responsiveness					
	11	I often leave food on my plate at the end of a meal	0.57	+	0.04
	23	I often get full before my meal is finished	0.63	+	0.03
	30	I cannot eat a meal if I have had a snack just before	0.54	+	0.03
	31	I get full up easily	0.77	+	0.03
Emotional undereating					
	15	I eat less when I’m worried	0.81	+	0.02
	18	I eat less when I’m angry	0.69	+	0.03
	20	I eat less when I’m upset	0.79	+	0.02
	27	I eat less when I’m annoyed	0.72	+	0.02
	35	I eat less when I’m anxious	0.84	+	0.02
Food fussiness					
	2	I often decide that I don’t like a food, before tasting it	0.64	+	0.03
	7	I refuse new foods at first	0.77	+	0.02
	12*	I enjoy tasting new foods	0.90	+	0.01
	19*	I am interested in tasting new food I haven’t tasted before	0.86	+	0.01
	24*	I enjoy a wide variety of foods	0.73	+	0.02
Slowness in eating					
	14*	I often finish my meals quickly	0.81		0.02
	25	I am often last at finishing a meal	0.88	+	0.01
	26	I eat more and more slowly during the course of a meal	0.62	+	0.03
	29	I eat slowly	0.92	+	0.01

*Reverse coded items.

**TABLE 4:** Confirmatory factor analysis results of two models of the adult eating behavior questionnaire in a sample of canadian adults (*n* = 534).

Model	Items	Factors	χ^2^ (df)	χ^2^/df	PR >χ^2^	CFI	GFI	RMSEA (95% CI)	AIC
(1)	35	8 Factors (H and FR items loaded separately)	1382.976 (532)	2.599	<0.001	0.908	0.864	0.055 (0.051, 0.058)	1578.976
(2)	35	7 Factors (H and FR items merged)	1537.917 (539)	2.853	<0.001	0.892	0.848	0.059 (0.056, 0.062)	1719.916
(3)	30	7 Factors (H items/factor deleted)	1036.203 (384)	2.698	<0.001	0.924	0.876	0.056 (0.052, 0.061)	1198.203

In accordance with our hypothesis, Food Approach subscales had positive inter-correlations (Table 5). In general, all Food Avoidance inter-correlations were positive and significant, except between Emotional Undereating and Food Fussiness (*r* = −0.03, *p* = 0.526) and between Food Fussiness and Slowness in Eating (*r* = −0.03, *p* > 0.05).

**TABLE 5:** Person’s correlations between the eight original AEBQ subscales and body mass index (BMI), *n* = 534.

		H	FR	EOE	EF	SR	EUE	FF	SE	BMI	BMI^a^
Food approach subscales	H	1.00	0.45**	0.24**	0.19**	−0.09*	0.003	−0.06	−0.05	−0.03	0.02
	FR		1.00	0.40**	0.48**	−0.35**	−0.14*	−0.09*	−0.30**	0.12*	0.19**
	EOE			1.00	0.19**	−0.16*	−0.57**	0.00	−0.19**	0.30**	0.34**
	EF				1.00	−0.28**	−0.14*	−0.26**	−0.16*	0.06	0.09*
Food avoidance subscales	SR					1.00	0.23**	0.16*	0.39**	−0.12*	−0.09*
	EUE						1.00	−0.03	0.21**	−0.28**	−0.26**
	FF							1.00	−0.03	0.07	0.06
	SE								1.00	−0.19**	−0.17**

^a^Analysis adjusted for age and sex. *p < 0.05, **p < 0.001. Subscales: EF, enjoyment of food; EOE, emotional overeating; FR, food responsiveness; H, hunger; EUE, emotional undereating; FF, food fussiness; SE, slowness in eating; SR, satiety responsiveness.

### Adult Eating Behavior Questionnaire Relationship With Three-Factor Eating Questionnaire-R18

Mean scores from the TFEQ-R18 were 2.32 ± 0.77 for Cognitive Restraint, 2.31 ± 0.60 for Uncontrolled Eating, and 2.23 ± 0.78 for Emotional Eating (data not shown). Correlations between Cognitive Restraint and AEBQ scores were non-significant, except for small positive correlations with Emotional Overeating (*r* = 0.16, *p* < 0.001), Satiety Responsiveness (*r* = 0.09, *p* = 0.03), and Slowness in Eating (*r* = −0.09, *p* = 0.04) (Table 6). In general, similar patterns of weak correlations were seen with mean scores from the Uncontrolled Eating and Emotional Eating scales with the AEBQ subscales. Some exceptions include the correlations between Uncontrolled Eating and Food Responsiveness (*r* = 0.72, *p* < 0.001), and Uncontrolled Eating with Food Responsiveness + Hunger merged subscale (*r* = 0.70, *p* < 0.001), indicating a strong correlation. The Emotional Eating scales also strongly correlated with Emotional Overeating (*r* = 0.82, *p* < 0.001).

**TABLE 6:** Pearson’s correlations between the eight original subscales and new variable (Food Responsiveness + Hunger) subscale with the Three-Factor Eating Questionnaire (TREQ-R18) (*n* = 534).

Pearson Correlation Coefficients									

	EF	EOE	FR	H	FR + H	EUE	FF	SE	SR
TFEQ-UE	0.31**	0.45**	0.72**	0.48**	0.70**	−0.20**	−0.00	−0.30**	−0.35**
TFEQ-CR	0.05	0.16*	0.06	−0.05	−0.00	−0.06	0.01	−0.09*	0.09*
TFEQ-EE	0.16*	0.82**	0.44**	0.27**	0.41**	−0.51**	0.04	−0.19**	−0.13*

*p < 0.05, **p < 0.001.

*AEBQ subscales:*

*EF, enjoyment of food; EOE, emotional overeating; FR, food responsiveness; H, hunger; EUE, emotional undereating; FF, food fussiness; SE, slowness in eating; SR, satiety responsiveness.*

*TFEQ-R18 subscales:*

*UE, uncontrolled eating; CR, cognitive restraint; EE, emotional eating.*

### Adult Eating Behavior Questionnaire Association With Body Mass Index

When the data was adjusted by age and sex (Table 2), participants classified to the overweight/obese categories had significantly higher subscale scores for Emotional Overeating (*p* < 0.001), Food Responsiveness (*p* < 0.001), and significantly lower scores for Emotional Undereating (*p* < 0.001) and Slowness in Eating (*p* = 0.001). When data were adjusted for BMI category and sex, comparisons of mean AEBQ subscales across age categories showed that participants aged 18–35 y had significantly higher subscale scores for Enjoyment of Food (compared to 56 y +, *p* < 0.001), Emotional Overeating (compared to 56 y +, *p* = 0.025), Food Responsiveness (compared to 36–55 y, *p* < 0.001 and compared to 56y +, *p* < 0.001), Hunger (compared to 36–55 y, *p* = 0.013 and compared to 56y +, *p* < 0.001), and Emotional Undereating (compared to 36–55 y, *p* = 0.029) (Table 2).

As expected, correlations between BMI (based on self-reported height and weight) and mean Food Approach subscales were positive, except for Hunger (*r* = −0.03, *p* = 0.423) and for Enjoyment of Food (*r* = 0.06, *p* > 0.05) (Table 5). Similarly, negative correlations were detected between BMI and Food Avoidance subscales, except for Food Fussiness (*r* = 0.07, *p* = 0.098). These results were consistent when adjusting for age and sex with the non-significant relationship between Enjoyment of Food and BMI becoming significant (*r* = 0.09, *p* < 0.05).

## Discussion

The primary aim of the present study was to confirm the fit statistics of different AEBQ models using the CFA, specifically analyzing for improvement of fit if the Hunger subscale was either merged with Food Responsiveness (Model 2) or removed from the questionnaire (Model 3). As described by Hunot et al. (2016), the Hunger subscale was intended to measure physical hunger (e.g., physical sensations in the body) knowing that perception of hunger differs among individuals (Wardle, 1987). Further, participant satiety status can potentially change how an individual answers questions related to hunger (Gibbons et al., 2019).

The metrics used to determine the goodness of fit from the CFA resulted in relatively decent model fit for all three models (Hu and Bentler, 1999). In line with the CFA findings of Mallan et al. who tested the AEBQ’s validity in an Australian sample (*n* = 998), the current study also found an improved fit statistic (e.g., smaller AIC) when the Hunger subscale was removed from the CFA (Mallan et al., 2017). As described by Mallan et al., these differences may be attributed to the differences in sample characteristics. These similar findings were also found in other validation studies conducted in adolescents (Hunot-Alexander et al., 2019) and Mexican adults (Hunot-Alexander et al., 2021a). Future studies require recruiting a diverse, gender balanced sample to continue to test the validity of the 7-factor versus the original 8-factor AEBQ among adult, especially as cultural differences may exist when describing relationships between eating behaviors and BMI (He et al., 2021). However, a recent study by Jacob et al., whose results support the original 8-factor over the 7-factor model, highlight that the hunger scale may be more related to characterizing maladaptive forms of eating regulation which are not associated with the risk of obesity (Jacob et al., 2021). As suggested by others, perception of hunger sensations differ across individuals (Zickgraf and Rigby, 2019; He et al., 2021), and that hunger may be regarded as a state rather than a trait (Hunot-Alexander et al., 2021a). Controlled feeding trials are needed to test the association between dietary intake, eating disorders and the AEBQ-hunger subscale to address these conflicting findings (Jacob et al., 2021).

The second aim of this study was to test for associations between AEBQ subscales and TFEQ-R18. We hypothesized that Food Approach scores would positively correlate with the Uncontrolled Eating and Emotional Eating TFEQ-R18 subscales. In particular, the AEBQ Food Approach subscales had moderate associations with TREQ-R18 Uncontrolled Eating (*r* = 0.45, *p* < 0.001) and strong associations with the TFEQ-R18 Emotional Eating scores (*r* = 0.82, *p* < 0.001). These findings are similar to those of He et al. (2021) supporting the discriminant validity of the full AEBQ scale, but some potential convergent validity of the AEBQ subscales in relation to the TFEQ-R18.

The third aim of this study was to explore the correlation of AEBQ and BMI. In this study, we found a pattern similar to the original publication where all Food Approach subscales were positively and significantly correlated with BMI with and without adjustments for age and sex (Hunot et al., 2016). Similar findings were found in a study that validated the AEBQ in a French-speaking Quebec population whereby weight and height were measured; individuals with overweight or obesity had higher scores for emotional overeating (*p* = 0.0002) and lower scores for emotional undereating (*p* = 0.02) (Jacob et al., 2021). These findings are contrary to others (Mallan et al., 2017; Zickgraf and Rigby, 2019) where higher BMI was not associated with all Food Approach subscales. As mentioned by Mallan et al., discrepant findings are likely due to the fact that BMI was self-reported in the majority of studies and therefore is less reliable than measured weight and height by a researcher. Nonetheless, our study contributes to the ongoing literature suggesting that the Food Approach scales are generally associated with higher BMI.

While this study was a cross-sectional study, one strength of this study was that it focused on testing important psychometric properties of the AEBQ and compares and contrasts all three models proposed in existing literature to provide evidence for the superiority of the 7-factor model in a Canadian sample. Future work should continue to test the use of the AEBQ in clinical settings as to our knowledge no work has reported on cut-off points related to scores of the AEBQ. To date, the tool has been validated in bariatric surgery candidates (Zickgraf and Rigby, 2019) against the eating habit sections of the Weight and Lifestyle Inventory, and in adolescents at risk of binge eating when receiving weight managing treatment (Molitor et al., 2021). Further, a recent study using the AEBQ to inform a personalized approach to weight management (the “Appetitive Trait Tailored Intervention”; ATTI) used cut-offs of above three as “high” for food approach traits and below three as “low” for food avoidance traits. This is the first time the AEBQ has been used in this way, and preliminary testing suggested the majority of participants found the intervention helpful (Hunot-Alexander et al., 2021b). Future work should continue to test the use of the AEBQ, and appropriate cut-offs, in clinical, and population settings.

Our study has limitations. Firstly, we acknowledge that our fit statistics failed to reach acceptable and optimal levels and that comparing some fit indices (i.e., the AIC) across different item pools (i.e., when all items are not included) may not be appropriate. Our recruitment efforts were predominately done through a generated Listserv from the PERFORM Centre, suggesting our representativeness of our sample may be limited to those who are familiar with our study center. As a result, our population was predominantly white, middle-aged women who reside in homes from higher household incomes in greater Montreal, Quebec, Canada. The recruitment method posed another limitation as the lack of information on response rates limit the information on potential recruitment biases or response biases. In particular, the unequal participant sex distribution (76% female) may serve as evidence of non-response bias, where online survey respondents may differ from non-respondents and thus provide different responses. Studies have shown that non-response can contribute to underestimating health risks in online health behavior surveys and those of poorer health tend to avoid participating compared to those of better health (Kypri et al., 2011). Future studies should consider preventive steps to non-response and record response rate for a more accurate examination. In addition, this study reports on data that are self-reported (i.e., weight, height) and therefore may contain bias such as recall bias and social desirability bias. The majority of participants (*n* = 301, 56.4%) were classified as having a normal BMI. Future studies may want to validate this questionnaire in different ethnic groups, as well as age groups (i.e., older adults). Given our small sample of individuals over the age of 65 years (mean age 39.5 ± 16.4 years), generalizations among age groups should be made with caution and future studies should focus on this vulnerable population. Similarly, participants had to self-declare that they were not living with an eating disorder nor did they have a history of an eating disorder. It was not possible to confirm this given the virtual nature of this study. Lastly, our study used mean scores as compared to factor scores, which may lead to us overlooking potentially important weighting of items within a subscale. Additionally, we acknowledge that obesity etiology extends beyond balancing dietary intake and physical activity since genetics, environment, and socio-cultural influences also play a role in the development of overweight and obesity.

## Conclusion

The present study suggests that a 30-question, 7-subscale AEBQ with removal of the Hunger subscale would be the most suitable AEBQ structure for assessing appetitive traits in a general English-speaking Canadian adult population. Construct validity of the AEBQ was achieved as positive correlations were found between all Food Approach subscales and Uncontrolled Eating and Emotional Eating scores from the TFEQ-R18 (convergent validity) while an overall weak association exists between the AEBQ and the TFEQ-R18 (discriminant validity). Future validation studies are warranted to confirm these findings in samples with more diversity in gender, ethnicity, and socioeconomic status.

## Data Availability Statement

The original contributions presented in the study are included in the article/supplementary material, further inquiries can be directed to the corresponding author.

## Ethics Statement

The studies involving human participants were reviewed and approved by Concordia Research Ethics Board. The patients/participants provided their written informed consent to participate in this study.

## Author Contributions

TC, LK, RB, CH-A, and HP designed the study. TC implemented the study. LK analyzed the data. RB and CH-A participated in interpretation of the data. All authors contributed to writing of the manuscript, read and approved the final manuscript.

## Conflict of Interest

The authors declare that the research was conducted in the absence of any commercial or financial relationships that could be construed as a potential conflict of interest.

## Publisher’s Note

All claims expressed in this article are solely those of the authors and do not necessarily represent those of their affiliated organizations, or those of the publisher, the editors and the reviewers. Any product that may be evaluated in this article, or claim that may be made by its manufacturer, is not guaranteed or endorsed by the publisher.

## Abbreviations

AEBQ: adult eating behavior questionnaire
AIC: akaike information criterion
BMI: body mass index.
